# Potential Exposure Characteristics and Self-Reported Symptom Burden in a Romanian Region with Heterogeneous Arsenic-Related Drinking-Water Risk

**DOI:** 10.3390/jox16040152

**Published:** 2026-08-15

**Authors:** Dana Claudia Filipoiu, Daniela Gitea, Manuela Bianca Pasca, Gabriela S. Bungau, Ada Radu, Raul Ștefan-Pantiș, Alin Mogoș, Bogdan Uivaraseanu, Delia Mirela Tit

**Affiliations:** 1 Doctoral School of Biological and Biomedical Sciences, University of Oradea, 410073 Oradea, Romania; filipoiu.danaclaudia@student.uoradea.ro (D.C.F.); gbungau@uoradea.ro (G.S.B.); dtit@uoradea.ro (D.M.T.); 2 Department of Pharmacy, Faculty of Medicine and Pharmacy, University of Oradea, 410028 Oradea, Romania; 3 Research Center for Thermal Analysis in Environmental Problems, ICAM-Advanced Environmental Research Institute, West University of Timisoara, Oituz Street 4, 300233 Timisoara, Romania; raul.stefan00@e-uvt.ro (R.Ș.-P.); alin.mogos@gmail.com (A.M.); 4 Doctoral School of Exact Sciences and Natural Sciences, West University of Timisoara, Pestalozzi Street 16, 300115 Timisoara, Romania; 5 Department of Surgical Disciplines, Faculty of Medicine and Pharmacy, University of Oradea, 410073 Oradea, Romania; buivaraseanu@uoradea.ro

**Keywords:** arsenic, drinking water, potentially toxic elements, human-health risk assessment, potential exposure index, symptom burden, environmental epidemiology

## Abstract

Environmental contamination by potentially toxic elements remains a public health concern in groundwater-dependent regions. This exploratory community-based cross-sectional study examined associations among a historical area-level arsenic screening classification, questionnaire-derived potential-exposure indicators, and self-reported symptom count in 275 adults from 37 standardized locality clusters in Satu Mare County, Romania. The arsenic Health Risk Index (HRI) screening classification was derived entirely from drinking-water measurements collected in a preceding regional study during 2022–2024; no water samples or exposure biomarkers were obtained in the present survey. The questionnaire-derived potential exposure index was calculated as the unweighted sum of seven binary indicators covering residential, drinking-water, occupational, dental, and dietary characteristics (theoretical range, 0–7; observed range, 0–6) and was analyzed continuously. Symptom count (theoretical range, 0–8) was analyzed primarily using negative binomial regression with locality-clustered standard errors and adjustment for age, sex, smoking, alcohol consumption, HRI classification, and self-reported comorbidity. Each one-point increase in the potential exposure index was associated with a modestly higher expected symptom count (adjusted incidence rate ratio = 1.106, 95% CI: 1.031–1.186; *p* = 0.005), whereas the area-level HRI classification was not statistically associated with symptom count (adjusted incidence rate ratio = 1.135, 95% CI: 0.829–1.554; *p* = 0.431). The association remained positive across alternative index specifications, although the drinking-water-focused estimate was smaller and less precise. These findings represent exploratory associations between reported potential-exposure characteristics and nonspecific self-reported symptoms; they do not establish individual arsenic exposure, contaminant-specific effects, or causality.

## 1. Introduction

Environmental contamination by potentially toxic elements remains a major public health challenge worldwide [1]. Due to their persistence in the environment, limited biodegradability, and capacity to accumulate in ecosystems and food chains, these elements may contribute to long-term human exposure through multiple pathways, including drinking water, food, occupational activities, and environmental pollution [2,3]. Among these contaminants, arsenic has received particular attention because of its widespread occurrence in groundwater and its documented association with adverse health outcomes under sufficiently high or prolonged exposure conditions [4,5].

Drinking water is recognized as one of the most important routes of human exposure to arsenic and other trace elements. Naturally occurring geogenic contamination of groundwater affects numerous regions worldwide, particularly areas relying on decentralized water systems and private wells [6,7,8]. Chronic exposure to arsenic-contaminated drinking water has been associated with dermatological and carcinogenic effects [9], neurological manifestations [10,11,12], cardiovascular disease [13,14], metabolic disorders [15], and a broader range of adverse health outcomes [16,17,18]. However, environmental contamination rarely occurs in isolation, and the health consequences observed at population level are often influenced by multiple environmental, behavioral, occupational, and individual health-related factors.

Assessment of environmental human-health risks traditionally relies on environmental monitoring data and risk assessment models. Health Risk Indices (HRIs) derived from contaminant concentrations in environmental media provide valuable information regarding potential non-carcinogenic health concerns at the screening level; however, area-level indicators may not fully capture variability in individual exposure [19]. Actual exposure depends on multiple factors, including water source, water consumption patterns, residential characteristics, occupational exposures, lifestyle behaviors, and other determinants that may modify cumulative exposure potential [20,21]. Consequently, integrating environmental monitoring findings with individual exposure-related characteristics has been increasingly recognized as an important approach in environmental epidemiology and public health research [22].

At the molecular level, the toxicity of arsenic is largely mediated by the generation of reactive oxygen species (ROS) and the resulting disruption of cellular redox homeostasis. Trivalent arsenic species exhibit high affinity for protein thiol groups and can inhibit antioxidant enzymes and sulfhydryl-dependent pathways, leading to oxidative stress, lipid peroxidation, and oxidative damage to proteins and nucleic acids [23]. Neural tissue appears particularly susceptible to this oxidative imbalance, and arsenic-induced oxidative stress has been proposed as a central mechanism underlying the peripheral neuropathy and sensory disturbances reported in chronically exposed populations [24]. These molecular processes provide biological context for the neurological and sensory manifestations described in populations with confirmed chronic arsenic exposure.

Although studies of chronically exposed populations have established important arsenic-related health effects, fewer investigations have examined how historical regional monitoring classifications correspond with current participant-reported drinking-water, residential, occupational, and lifestyle characteristics. Environmental monitoring and questionnaire-derived information capture analytically different dimensions: the former characterizes sampled environmental media, whereas the latter describes reported circumstances that may influence potential contact but does not quantify internal exposure. Evaluating their correspondence may help clarify the informational value and limitations of combining these data sources in exploratory community-based environmental-health research.

In a previous regional investigation conducted in Satu Mare County, Romania, our research group evaluated concentrations of arsenic and other potentially toxic elements in water intended for human consumption and estimated associated non-carcinogenic health risks using United States Environmental Protection Agency methodology. Although most measured elements were within acceptable limits, substantial spatial variability was observed for arsenic concentrations, with several localities, particularly rural communities relying on decentralized groundwater systems, exhibiting elevated arsenic-related Health Risk Indices [25]. These findings raised important questions regarding whether locality-level environmental risk patterns are reflected in resident-level potential-exposure characteristics and whether such characteristics are associated with self-reported health outcomes.

The present study was designed as an exploratory community-based cross-sectional investigation of whether a historical area-level arsenic screening classification and questionnaire-derived potential-exposure characteristics were associated with self-reported symptom count among adults in Satu Mare County. The analysis was intended to generate hypotheses and assess the complementary information provided by area-level environmental screening patterns, without inferring current individual arsenic exposure or causal health effects.

## 2. Materials and Methods

### 2.1. Study Design and Participants

**Figure 1:**
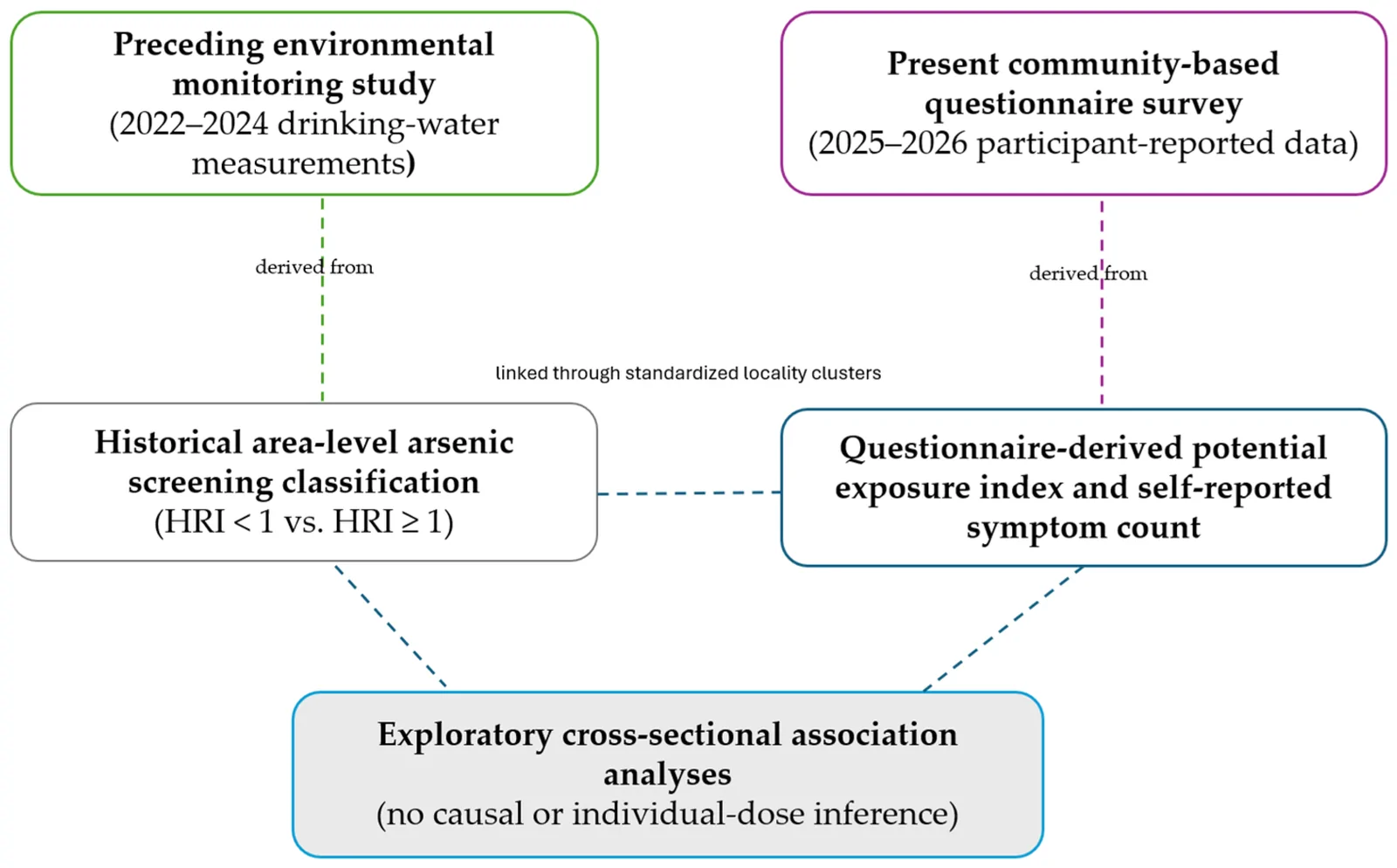
Analytical framework distinguishing the preceding sample-level environmental dataset, the present questionnaire data, and the exploratory statistical associations examined. Dashed lines indicate data linkage or statistical association only and do not represent causal pathways.

This exploratory community-based cross-sectional association study included adults residing in Satu Mare County, Romania. It was designed as a questionnaire-based extension of a preceding regional investigation of PTEs in water intended for human consumption [25]. The primary objective was to examine the association between a potential exposure index and self-reported symptom count. A secondary objective was to assess the relationship between a historical area-level arsenic screening classification, reported potential-exposure characteristics, and symptom count. The analytical framework is shown in Figure 1.

Eligibility criteria were age ≥ 18 years, residence in Satu Mare County, ability to understand and complete the Romanian-language questionnaire, and provision of written informed consent. Individuals younger than 18 years and those who declined participation were not enrolled. Questionnaires missing one or more variables required for the complete-record analyses were excluded from the analytical sample.

Recruitment was conducted from November 2025 through May 2026 using a convenience-based community approach in urban and rural areas of Satu Mare County. Paper questionnaires were distributed through local institutional partners, including family physicians and local administrative authorities. Participation was voluntary, and questionnaires were completed after written informed consent. Recruitment was neither probability-based nor consecutive.

Of the 500 questionnaires distributed, 302 were returned, corresponding to a questionnaire return rate of 60.4%. Twenty-seven questionnaires were excluded because one or more variables required for the complete-record analyses were missing, leaving 275 participants in the final analytical sample. Numbers distributed and returned were not recorded separately by locality; therefore, locality-specific response rates and comparisons between respondents and nonrespondents could not be calculated. Final analyzed counts for the 37 standardized locality clusters are reported in Supplementary Table S1.

Participants were assigned to 37 standardized locality clusters. For the present analysis, participants were classified as HRI ≥ 1 when their reported locality of residence corresponded to a locality in which the preceding study had identified at least one drinking-water sample with an adult arsenic HRI > 1 [25]. Participants residing in the remaining included localities were classified as HRI < 1. Where repeated samples or multiple source-study records referred to the same locality, the locality was assigned to HRI ≥ 1 if at least one adult sample exceeded the HRI threshold. The classification was harmonized across the standardized locality clusters, resulting in seven clusters classified as HRI ≥ 1 and 30 classified as HRI < 1.

**Figure 2:**
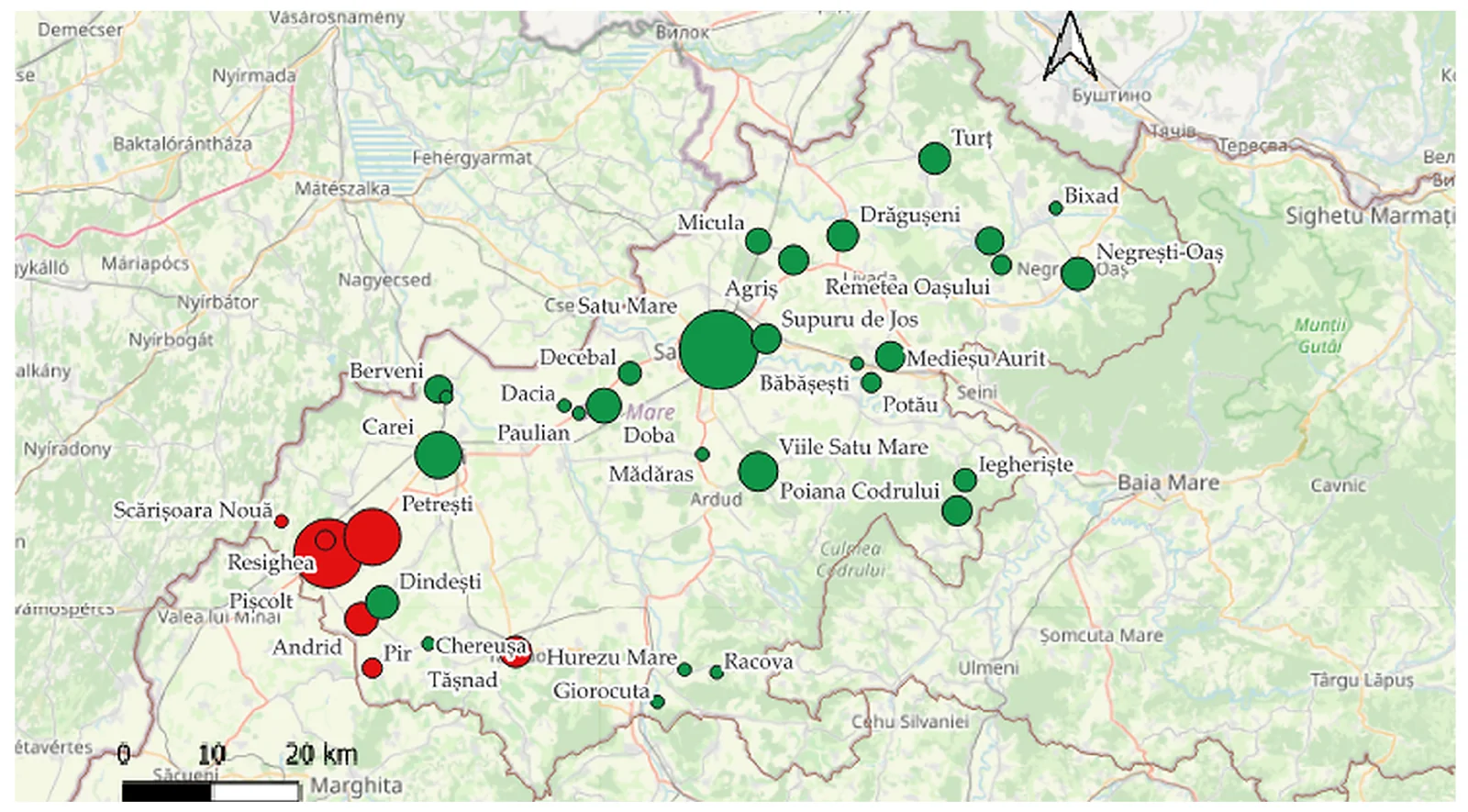
Geographic distribution of analyzed participants and the historical area-level arsenic screening classification used for participant linkage in Satu Mare County. Circle size is proportional to the analyzed participant count. Red circles indicate clusters assigned to the historical HRI ≥ 1 category, and green circles indicate clusters assigned to HRI < 1. The classification was derived from the preceding environmental dataset and represents an area-level historical screening context; it does not indicate participant-specific water concentration, exposure, or dose. Generated using QGIS version 3.40.14, with the Esri basemap accessed through ArcGIS Online [26].

Because some sampling labels in the source study represented shared water-supply systems or combined geographic areas, the classification was treated exclusively as a historical area-level linkage variable. It indicated that at least one sample-level exceedance had previously been identified in the relevant locality or standardized cluster; it did not imply that all local water sources were affected or that any participant consumed water from the sampled source. No measured arsenic concentration, household-water value, individual exposure, or dose was assigned to a participant. The geographic distribution of the analyzed sample and the historical screening classification are shown in Figure 2.

No formal a priori sample-size calculation was performed because the study was exploratory and recruitment was based on the number of completed questionnaires available within the study period. The demographic composition of the analytical sample was compared descriptively with the 2021 Satu Mare County census. The sample included higher proportions of women (61.5% vs. 51.6%) and rural residents (68.0% vs. 56.1%) and had a higher mean age (49.6 vs. approximately 41 years) than the county population [27]. The age comparison is approximate because the survey included adults only, whereas the county mean includes all age groups. These comparisons are descriptive and do not establish representativeness.

The questionnaire study was conducted within the scope of the broader doctoral research project approved by the Ethics Committee of the University of Oradea (Approval No. CEFMF/02, 29 July 2021). All participants provided written informed consent.

### 2.2. Questionnaire Development and Data Collection

Data were collected using a structured questionnaire developed for this exploratory study to document sociodemographic characteristics, participant-reported potential environmental and occupational exposure characteristics, lifestyle factors, selected self-reported symptoms and physician-diagnosed conditions, and perceptions of drinking-water quality. General principles of questionnaire development and content validity were considered during questionnaire design [28,29]. Item selection was informed by literature on arsenic exposure assessment and health effects [8,30,31], environmental and occupational exposure pathways and environmental-health risk assessment [32,33,34], mercury exposure from dental amalgam [35], and dietary exposure associated with fish consumption [36], together with findings from the preceding regional monitoring study [25].

The draft questionnaire underwent qualitative content review by a multidisciplinary panel with expertise in toxicology, pharmacy, public health, and research methodology. Panel members assessed item relevance, domain coverage, scientific appropriateness, wording clarity, and comprehensibility. Items considered unclear, ambiguous, redundant, or insufficiently relevant were revised or removed, and the final wording was agreed upon by consensus. No quantitative content-validity index was calculated. No formal cognitive interviews, pilot psychometric study, or construct-validity analyses were conducted.

The questionnaire was administered in Romanian. The English version provided in Supplementary Material S1 was prepared solely to facilitate transparent reporting and was not used for data collection. Accordingly, no cross-cultural validation of the English reporting version was undertaken. The questionnaire was intended to characterize participant-reported potential-exposure characteristics and nonspecific symptom burden (Table 1). It was not designed to establish clinical diagnoses, attribute symptoms to arsenic or another contaminant, quantify exposure intensity, or estimate individual internal exposure or dose.

### 2.3. Historical Environmental Context, Potential Exposure Index, and Symptom Outcomes

Two analytically distinct data sources were used: (i) sample-level environmental measurements and risk calculations reported in the preceding study [25] and (ii) participant responses collected in the present questionnaire survey. The preceding environmental investigation included 271 drinking-water samples collected from 122 localities during 2022–2024: 181 samples from centralized public distribution systems, 15 from private sources, including private wells, two from system entry or exit points, and 73 records with unspecified source type. Arsenic results were available for 244 samples and ranged from 0.075 to 320.50 µg/L. A summary of the published multi-element analytical profile, including concentration ranges, applicable drinking-water limits, and interpretive context for As, Cd, total Cr, Mn, Ni, Pb, and Se, is provided in Supplementary Table S2. In the preceding study, adult chronic daily intake was calculated separately for each sample as CDI = C × IR/BW, where C was the measured arsenic concentration in mg/L, IR was 2 L/day, and BW was 70 kg. Adult arsenic HRI was calculated as CDI/RfD using the oral RfD of 0.0003 mg/kg/day applied in that study [25,32]. All HRI values were generated in the preceding study at the individual-sample level and were not recalculated from newly collected environmental data in the present survey. Fourteen of the 244 arsenic measurements had an adult HRI > 1.

For the present questionnaire analysis, participants were linked by standardized locality cluster to the binary historical screening classification described above. Localities in which the preceding study had identified at least one adult sample with HRI_As > 1 were assigned to the HRI ≥ 1 category; the remaining localities were assigned to HRI < 1. Duplicate sample-level records were retained in the source study, but the present classification was binary at locality-cluster level. No sample-level concentration, household-water measurement, or individual dose was assigned to a participant.

The 2025 US EPA IRIS assessment subsequently established an inorganic-arsenic oral RfD of 0.00006 mg/kg/day [37]. The historical classification was retained to preserve consistency with the methodology and screening context of the preceding study and was not interpreted as a current risk classification under the updated RfD.

Environmental sampling occurred during 2022–2024, whereas questionnaire administration occurred from November 2025 through May 2026, producing an interval of approximately one to four years. No repeated household-level measurements were available to establish that water sources or arsenic concentrations remained stable during this period. Furthermore, residents assigned to the same locality cluster reported heterogeneous primary drinking-water sources. The HRI variable was therefore used solely as a historical area-level screening indicator and not as an estimate of current individual arsenic exposure. The environmental monitoring inputs, source-study HRI calculations, participant-linkage approach, and their interpretation in the present study are summarized in Table 2.

A questionnaire-derived potential exposure index (hereafter, the potential exposure index) was constructed as the unweighted sum of seven binary indicators representing reported residential, drinking-water, occupational, dental, and dietary characteristics: well-water use, rural residence, daily water intake ≥ 2 L, occupational contact with chemical agents, occupational exposure to dust or smoke, currently present dental amalgam restorations, and fish consumption ≥ 3 times/week. Equal weighting was adopted as a pragmatic counting approach because no participant-level contaminant measurements, biomonitoring data, toxicokinetic coefficients, or externally validated weights were available to support differential weighting. The index therefore represents the number of reported potential-exposure characteristics and does not imply equivalence among components in exposure intensity, dose, chemical form, toxicological potency, or health risk.

The potential exposure index had a theoretical range of 0–7 and an observed range of 0–6 and was analyzed continuously. It was treated as an exploratory formative indicator and was not validated against environmental measurements or biomarkers. To evaluate sensitivity to index construction, four alternative specifications were examined: (i) an alternative weighted specification assigning three points to well-water use, two points to rural residence, and one point to each of the remaining five indicators; (ii) a drinking-water-focused specification comprising well-water use and daily water intake ≥ 2 L; (iii) an equally weighted index excluding rural residence; and (iv) an equally weighted index excluding dental amalgam and frequent fish consumption. Because these specifications had different numerical ranges, their associations were expressed per one population-standard-deviation increase, calculated using the sample mean and population standard deviation. An additional negative binomial model entered all seven binary components simultaneously, together with the same adjustment set used in the primary model.

Symptom burden was operationalized as a count of eight binary self-reported items: allergies or intolerances, skin problems, metallic taste, numbness or tingling sensations, memory or concentration difficulties, sleep disturbances, frequent headaches, and irritability or mood changes. Positive responses were summed to produce a symptom count with a theoretical range of 0–8 and an observed range of 0–7. Symptom count was analyzed as the primary outcome. For a secondary sensitivity analysis, symptom count was dichotomized as <3 versus ≥3 symptoms. This threshold was used only as an analytical grouping and has no validated clinical or diagnostic interpretation.

The coding and analytical interpretation of the historical area-level screening classification, potential exposure index, symptom count, and binary symptom outcome are summarized in Table 3.

### 2.4. Data Management and Statistical Analysis

Questionnaire responses were entered into a standardized database and independently checked for completeness, coding accuracy, implausible values, and consistency between source and derived variables. Locality names were standardized before participant clustering. The potential exposure index, alternative index specifications, symptom count, binary symptom outcome, and any-comorbidity indicator were generated using reproducible code. All 275 records were complete for the variables included in the statistical models.

Descriptive and nonparametric analyses were performed using JASP version 0.19.3. Regression models, cluster-robust inference, model diagnostics, and sensitivity analyses were conducted in R using RStudio—R version 4.6.0 (2026-04-24). Continuous variables are presented as mean ± standard deviation and median with interquartile range, and categorical variables as n (%). Distributional characteristics were assessed descriptively and using the Shapiro–Wilk test.

Associations involving ordinal or non-normally distributed variables were examined using nonparametric methods. Differences between two independent groups were evaluated using the Mann–Whitney U test, with rank-biserial correlation reported as an effect-size measure. Associations between continuous or ordinal variables were evaluated using Spearman’s rank correlation coefficient. Associations between categorical variables were examined using the chi-square test, with Cramér’s V reported as an effect-size measure. Symptom counts were compared across the four reported primary drinking-water sources using the Kruskal–Wallis test, followed by Dunn pairwise comparisons with Holm adjustment.

As a descriptive assessment of covariation among the eight symptom items, Cronbach’s α, its Feldt 95% confidence interval, the average inter-item correlation, and item–rest correlations were calculated. These statistics were not used to claim construct validity, unidimensionality, or diagnostic reliability because the items were intentionally heterogeneous and no psychometric validation study had been performed.

Exploratory associations between the potential exposure index and the eight individual binary symptom items were evaluated using separate logistic regression models with finite-sample-corrected standard errors clustered by locality. Holm-adjusted *p* values were calculated across the eight tests to control the family-wise error rate. Both unadjusted and Holm-adjusted *p* values are reported. These analyses were not used to infer a contaminant-specific effect, a validated symptom pattern, or a causal relationship.

The primary multivariable outcome was the complete symptom count. Poisson and negative binomial log-link models were compared after examination of the outcome variance, Pearson dispersion, and the estimated negative binomial dispersion parameter. A boundary likelihood-ratio test was used to compare the two specifications. Because the Poisson model showed overdispersion and the negative binomial model provided a better fit, the negative binomial specification was selected as the primary model. Finite-sample-corrected sandwich standard errors clustered by standardized locality were used to account for within-locality correlation across the 37 clusters.

The prespecified adjustment set included the potential exposure index, age per 10 years, sex, smoking status (never, current, or former), alcohol consumption (none or any), historical area-level arsenic screening classification, and an any-comorbidity indicator. The latter was coded as positive when a participant reported at least one physician-diagnosed cardiovascular, diabetic, kidney, neurological, or thyroid condition. The individual diagnostic categories were combined because of their relatively low frequencies and to limit model instability and overparameterization. Results are reported as adjusted incidence rate ratios with 95% confidence intervals.

Model adequacy was evaluated using convergence status, the estimated negative binomial dispersion parameter, Pearson dispersion, Akaike information criterion, McFadden pseudo-R^2^, variance inflation factors, and joint likelihood-ratio testing of quadratic terms for age and the potential exposure index.

As an outcome-sensitivity analysis, a secondary locality-clustered logistic regression model was fitted using the analytical grouping of <3 versus ≥3 symptoms. This model included the same prespecified predictors as the primary negative binomial model and used finite-sample-corrected standard errors clustered by locality. Results are reported as adjusted odds ratios with 95% confidence intervals. Model performance was summarized using Nagelkerke R^2^, the Brier score, the Hosmer–Lemeshow goodness-of-fit test, and the area under the receiver operating characteristic curve, with a 1000-replicate locality-cluster bootstrap confidence interval. Linearity of age and the potential exposure index on the logit scale was assessed by comparing the main-effects model with an expanded model containing quadratic terms for both continuous predictors.

Sensitivity to index construction was evaluated using the four alternative index specifications described above. Because the indices had different numerical ranges, their associations were expressed per one population-standard-deviation increase. An additional negative binomial model entered all seven potential-exposure components simultaneously, together with the same covariate adjustment set used in the primary model. These analyses were exploratory and were intended to assess the dependence of the principal result on index construction rather than to identify a contaminant or exposure pathway.

Potential overlap between the historical HRI classification and the potential exposure index was assessed using variance inflation factors in the fully adjusted models. Because the HRI classification was a historical area-level variable and the index summarized participant-reported characteristics, their association was interpreted as contextual overlap rather than measurement of the same individual-level construct.

All tests were two-sided, and *p* < 0.05 was considered statistically significant. Multiplicity adjustment was applied to the eight symptom-specific models and to post hoc drinking-water-source comparisons. The remaining sensitivity and component-level analyses were exploratory, and their *p* values were not adjusted for multiple testing.

## 3. Results

### 3.1. Characteristics of the Study Population

A total of 275 participants from 37 standardized locality clusters were included in the analyses. The mean age was 49.6 ± 14.9 years (range: 19–86 years), and 61.5% of participants were women. Most participants resided in rural areas (68.0%), while 33.1% were assigned to the historical HRI ≥ 1 area-level screening category.

The most commonly reported drinking water sources were bottled water (30.2%) and well water (29.5%), followed by tap water (26.2%) and filtered water (14.2%). Approximately one-third of participants reported the presence of dental amalgam restorations (35.3%), and nearly one-quarter were current smokers (24.7%).

The questionnaire-derived potential exposure index had a mean of 2.20 ± 1.35 and a median of 2 points (IQR: 1–3; observed range: 0–6). The median symptom count was 1 (IQR: 0–3), and 82 participants (29.8%) reported ≥3 symptoms. Additional population characteristics are presented in Table 4.

Cronbach’s α for the eight symptom items was 0.618 (Feldt 95% CI: 0.545–0.682), and the average inter-item correlation was 0.169. Item–rest correlations ranged from 0.193 to 0.396, and deletion of individual items did not materially increase α. These results indicate modest covariation among the heterogeneous symptom items and are reported descriptively rather than as evidence of a validated unidimensional scale.

### 3.2. Historical Area-Level Arsenic Screening Classification and Participant-Reported Characteristics

**Figure 3:**
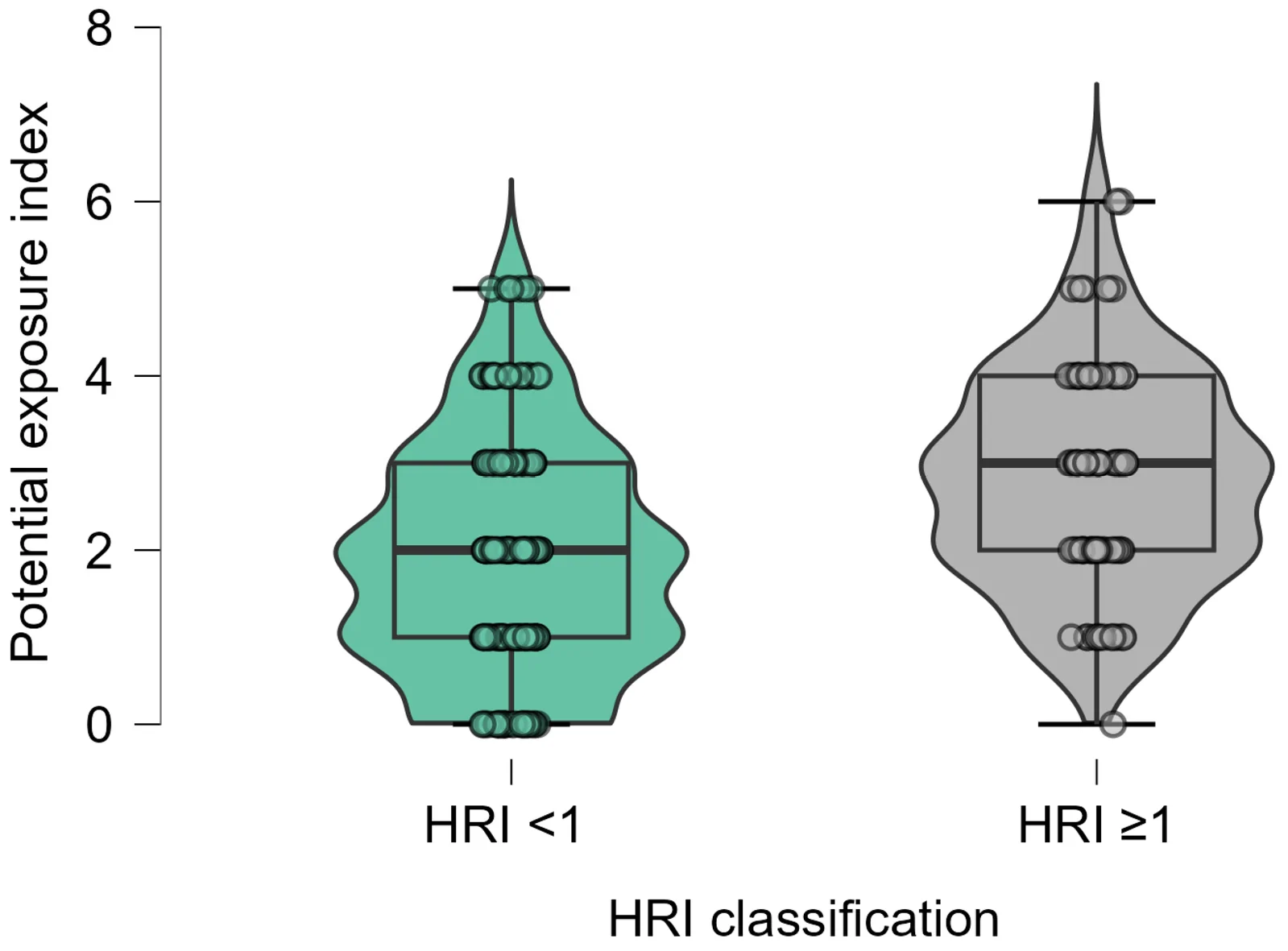
Potential exposure index according to the historical area-level arsenic screening classification. Participants assigned to the HRI ≥ 1 category had higher index values than those assigned to HRI < 1 (Mann–Whitney U = 5168.5, *p* < 0.001). The historical classification represents area-level contextual information and not participant-specific arsenic exposure.

Participants assigned to the historical HRI ≥1 category had higher potential exposure index values than those assigned to HRI < 1 (median: 3 [IQR: 2–4] vs. 2 [IQR: 1–3]; Mann–Whitney U = 5168.5, *p* < 0.001; rank-biserial r = 0.383; Figure 3). Mean index values were 2.81 ± 1.23 and 1.90 ± 1.31, respectively, and the historical classification was modestly correlated with the questionnaire-derived index (Spearman’s ρ = 0.319, *p* < 0.001).

Well-water use was also more frequent among participants assigned to HRI ≥ 1 than among those assigned to HRI < 1 (40.7% vs. 23.9%; χ^2^ = 8.218, *p* = 0.004; Cramér’s V = 0.173). Nevertheless, only 37 of the 91 participants assigned to HRI ≥ 1 reported well water as their primary source, whereas 44 participants assigned to HRI < 1 also reported well-water use. These findings indicate partial contextual overlap between the historical area-level classification and current participant-reported characteristics, but not agreement between two measures of individual exposure.

### 3.3. Unadjusted Association Between the Potential Exposure Index and Symptom Count

The potential exposure index was weakly correlated with symptom count (Spearman’s ρ = 0.205, *p* = 0.001). This unadjusted association reflects covariation between the number of reported potential-exposure characteristics and the number of nonspecific symptoms and should not be interpreted as a contaminant-specific dose–response relationship.

**Figure 4:**
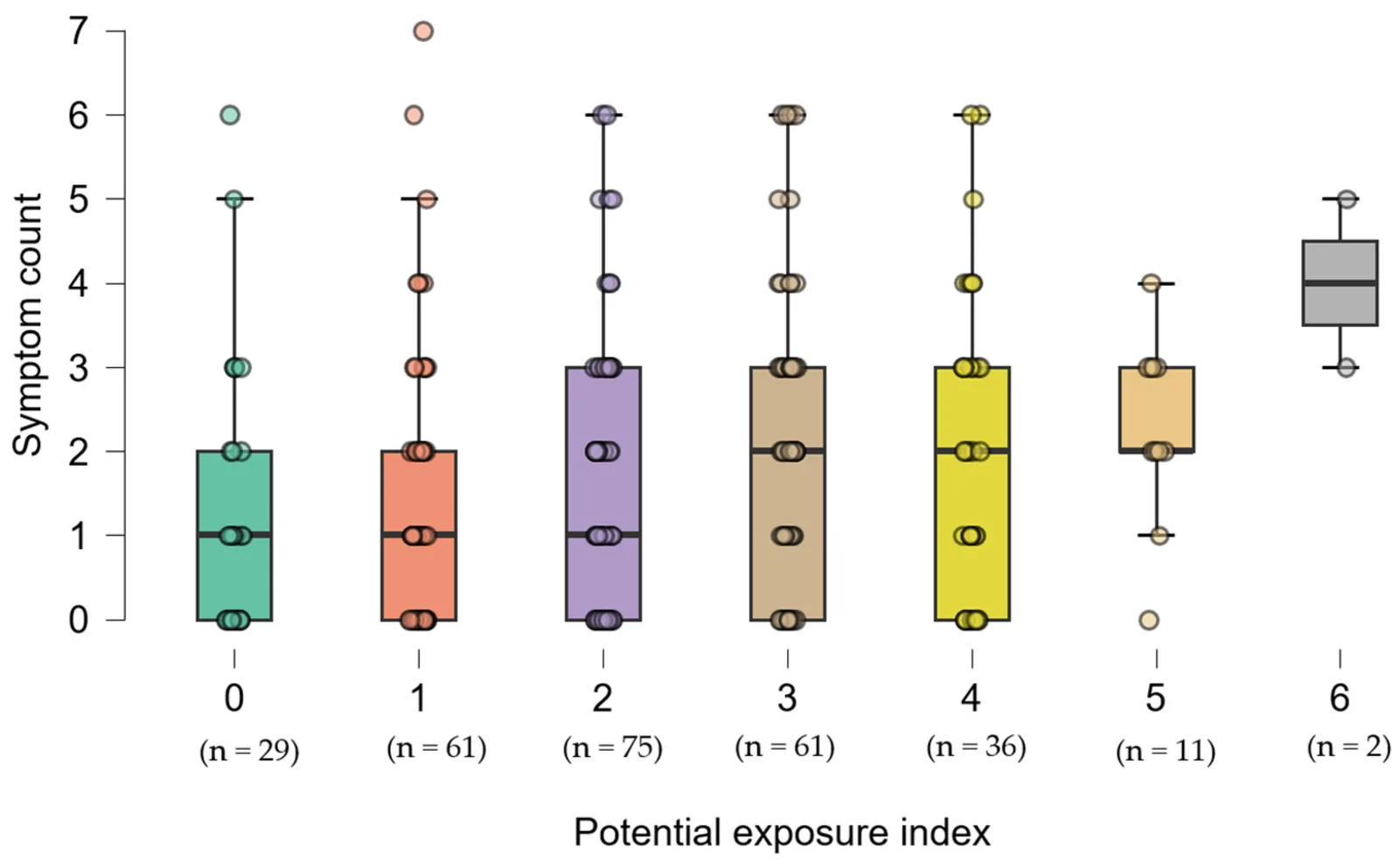
Self-reported symptom count across the exact observed values of the questionnaire-derived potential exposure index. Boxes show the median and interquartile range, individual observations are superimposed, and participant counts are displayed below the axis. The figure is descriptive; index values count reported potential-exposure characteristics and should not be interpreted as toxicological exposure levels or thresholds.

Figure 4 shows the observed symptom-count distribution at each exact index score. No low, moderate, or high groupings or score-level pairwise comparisons were defined. Because only 11 participants had a score of 5 and two had a score of 6, the score-specific distributions are presented descriptively and should not be interpreted as threshold effects.

The weak correlation and sparse observations at the highest scores support a cautious interpretation of the index as an exploratory summary of heterogeneous reported characteristics.

### 3.4. Drinking-Water Source and Symptom-Specific Exploratory Analyses

Symptom count did not differ between participants from HRI < 1 and HRI ≥ 1 localities (Mann–Whitney U = 7761.0, *p* = 0.312; rank-biserial r = 0.073).

**Figure 5:**
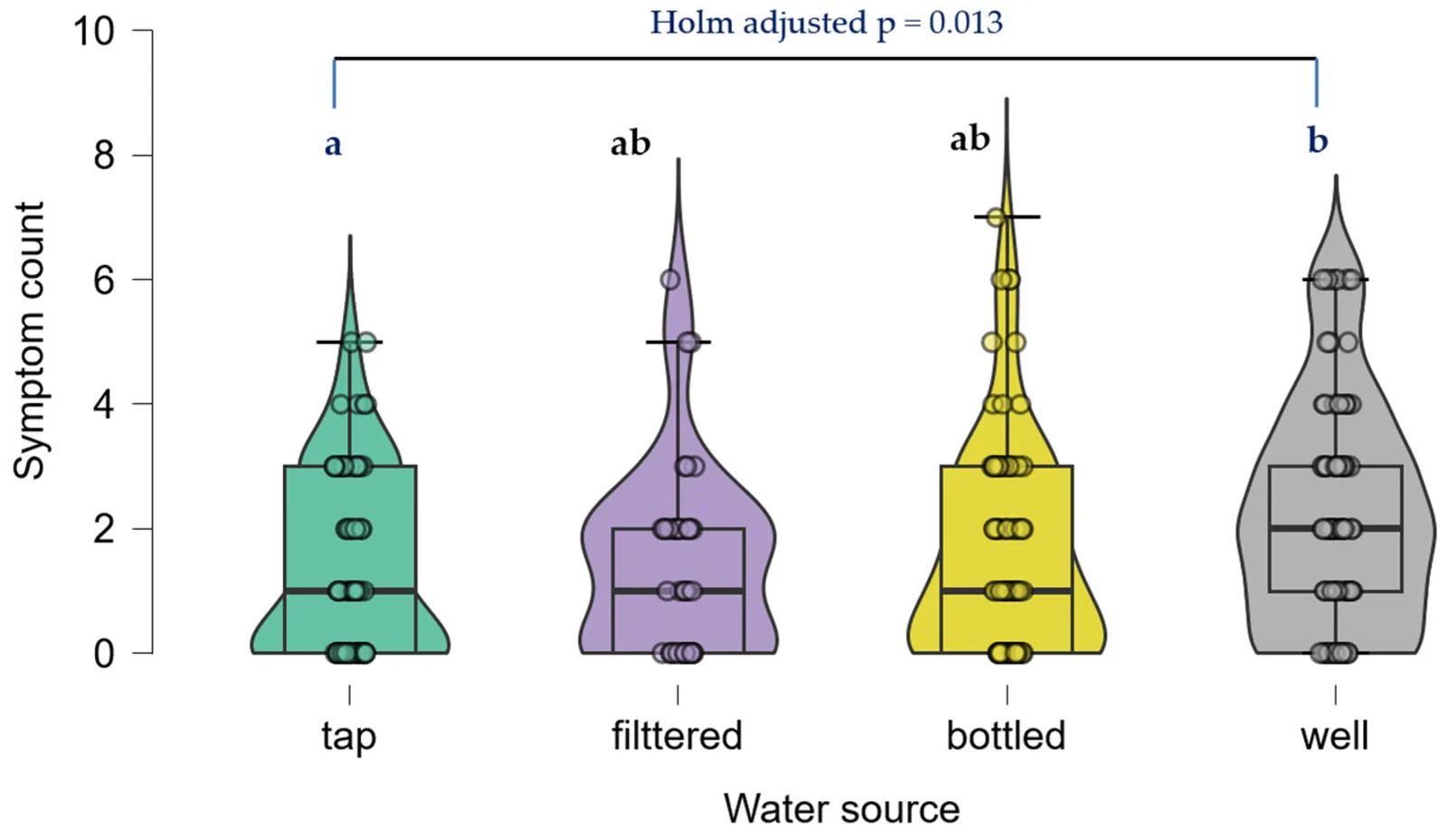
Self-reported symptom count according to the primary drinking-water source. The overall comparison was statistically significant (Kruskal–Wallis H = 11.881, *p* = 0.008). Compact letters indicate Dunn–Holm-adjusted pairwise comparisons; groups sharing a letter were not statistically different. Only well-water and tap-water users differed after adjustment (adjusted *p* = 0.013).

Symptom count differed across reported primary drinking-water sources (Kruskal–Wallis H = 11.881, *p* = 0.008; Figure 5). Well-water users had the highest mean count (2.22 ± 1.76), while tap-water users had the lowest (1.38 ± 1.48). Only the well-water versus tap-water comparison remained supported after Dunn–Holm correction (adjusted *p* = 0.013). Because no household water was tested, this comparison cannot identify the contaminant responsible for the observed difference.

Exploratory item-specific models were fitted to examine whether the association with the potential exposure index was concentrated in particular symptoms. After Holm correction across the eight tests, only metallic taste remained statistically supported (OR per point = 1.798, 95% CI: 1.223–2.642; adjusted *p* = 0.023). Nominal associations for skin problems and sleep disturbances did not remain statistically supported after Holm correction, and no association was observed for numbness or tingling (Table 5).

### 3.5. Primary Multivariable and Outcome-Sensitivity Analyses

Each one-point increase in the potential exposure index was associated with a 10.6% higher expected symptom count after adjustment for the measured covariates and locality clustering (adjusted IRR = 1.106, 95% CI: 1.031–1.186; *p* = 0.005; Table 6). Self-reported comorbidity showed the strongest association with symptom count (adjusted IRR = 1.798, 95% CI: 1.498–2.160; *p* < 0.001), and its inclusion materially improved model fit (likelihood-ratio χ^2^ = 22.568; *p* < 0.001). Higher expected symptom counts were also observed with increasing age, female sex, and any alcohol consumption. The historical area-level arsenic screening classification and both smoking indicators were not statistically supported. The Poisson model showed overdispersion (Pearson dispersion = 1.440), and the negative binomial specification provided a better fit (boundary likelihood-ratio χ^2^ = 7.233; *p* = 0.004). The final negative binomial model converged with α = 0.158, Pearson dispersion = 1.177, AIC = 909.870, and McFadden pseudo-R^2^ = 0.079. Joint quadratic terms for age and the potential exposure index did not improve model fit (*p* = 0.639), and the maximum VIF was 1.442, indicating no problematic multicollinearity among the modeled predictors.

In the logistic sensitivity model, each one-point increase in the potential exposure index was associated with higher odds of reporting at least three symptoms (adjusted OR = 1.349, 95% CI: 1.089–1.672; *p* = 0.006; Table 6). The model included 82 events and had an AUC of 0.740 (1000-replicate locality-cluster bootstrap 95% CI: 0.708–0.823), a Brier score of 0.179, and a Nagelkerke R^2^ of 0.199. The Hosmer–Lemeshow test provided no evidence of poor calibration (χ^2^(8) = 3.529; *p* = 0.897), and joint quadratic terms did not improve model fit (likelihood-ratio χ^2^(2) = 2.330; *p* = 0.312).

### 3.6. Alternative Index Specifications and Component-Level Analyses

The association between the potential exposure index and symptom count remained positive across the alternative index specifications (Table 7). When expressed per one population-standard-deviation increase, the adjusted IRR was 1.146 (95% CI: 1.043–1.259; *p* = 0.005) for the primary equally weighted index and 1.134 (95% CI: 1.033–1.244; *p* = 0.008) for the alternative weighted specification. Positive estimates were also observed for the index excluding rural residence and for the index excluding dental amalgam and frequent fish consumption. The drinking-water-focused specification produced a smaller and less precise estimate (adjusted IRR = 1.076, 95% CI: 0.992–1.166; *p* = 0.076).

When the seven components were entered simultaneously, well-water use and occupational dust/smoke exposure were associated with higher expected symptom counts, whereas the remaining component estimates were not statistically supported. These analyses were exploratory and were intended to assess the sensitivity of the principal finding to index construction rather than to identify a specific contaminant or exposure pathway.

## 4. Discussion

Environmental monitoring provides an essential basis for identifying geographic areas in which drinking-water contamination may warrant further attention, but it does not fully capture the variability of individual exposure circumstances. In the preceding regional study conducted in Satu Mare County, substantial spatial heterogeneity was observed in arsenic concentrations and related human-health screening indices, particularly in communities relying on decentralized groundwater sources [25]. Building on that environmental context, the present study examined how historical area-level screening information relates to current participant-reported residential, water-use, occupational, and lifestyle characteristics.

The findings showed only partial correspondence between the two levels of information. Participants assigned to the HRI ≥1 category had higher potential exposure index values and more frequently reported well-water use, indicating some overlap between the historical environmental context and current participant-reported circumstances. However, the historical area-level classification was not associated with symptom count, whereas the questionnaire-derived index showed a modest positive association after adjustment for measured demographic, behavioral, and health-related covariates and locality clustering.

These findings likely reflect the different information captured by the two approaches. Environmental measurements characterize contaminants at specific locations and time points, whereas questionnaires describe residential circumstances, water-use practices, occupational contacts, and lifestyle characteristics that may influence opportunities for exposure. Current exposure-assessment frameworks therefore support combining environmental measurements with information on individual activities and exposure factors and, where feasible, biomarkers of internal exposure [38].

The association observed for the potential exposure index should be interpreted in light of its formative construction. The index does not represent contaminant dose or a unified toxicological construct; rather, it summarizes the coexistence of several residential, drinking-water, occupational, dental, and dietary characteristics reported by participants. Its components are therefore not expected to be strongly correlated or to contribute equally in biological terms. The index should be interpreted strictly as a pragmatic exploratory summary of reported exposure-related characteristics rather than a quantitative environmental exposure metric.

The generally consistent direction of estimates across alternative specifications suggests that the association was not driven by a single component or weighting choice [39]. However, the smaller and less precise estimate for the drinking-water-focused specification, together with the heterogeneous component-level findings, does not support an interpretation centered specifically on drinking water or arsenic. Rather, the index appears to reflect a broader set of reported circumstances potentially relevant to environmental contact through different routes. Biomonitoring can integrate exposure across multiple sources and pathways but does not necessarily identify the source responsible for a measured internal concentration [40]. The questionnaire-derived index may therefore complement historical environmental information, but it cannot replace source-specific measurements or biological exposure assessment.

The absence of an association between the historical HRI classification and symptom count is understandable given the way environmental information was linked to participants. Source measurements were collected at specific locations during 2022–2024, whereas questionnaire data were obtained one to four years later, and no repeated household-level testing was available to confirm the stability of water sources or arsenic concentrations. Participants within the same locality cluster also reported different primary drinking-water sources, including bottled, filtered, tap, and well water. These spatial and temporal discrepancies may introduce exposure misclassification and limit the ability of an area-level historical classification to represent current personal exposure. Such measurement error can attenuate or otherwise distort associations in observational epidemiology, particularly when the underlying error structure cannot be characterized [41].

The change in the arsenic reference dose further supports interpreting the HRI classification as historical screening context rather than a current participant-level risk estimate. The preceding investigation calculated HRI using the oral reference dose applied at that time, whereas the 2025 US EPA IRIS assessment subsequently established a lower oral reference dose for inorganic arsenic [37]. The historical classification was retained to preserve methodological correspondence with the source study, not to reclassify present-day individual risk. More generally, screening indices depend on the exposure assumptions, reference values, and modeled pathways used in their derivation and must be interpreted within that assessment framework [42].

The wider source-study profile also documented repeated manganese exceedances and isolated maxima above the applicable limits for nickel and selenium, whereas cadmium, total chromium, and lead remained below their respective maximum admissible concentrations [25]. Because these measurements were not linked to participants through current source-specific data, they provide regional environmental context but cannot explain the symptom associations observed in the present survey.

The findings related to drinking-water source provide additional context. Participants reporting well water as their primary source had higher symptom counts than tap-water users, and well-water use remained positively associated with symptom count when all seven index components were considered simultaneously. Groundwater can contain naturally occurring arsenic and other chemical constituents, with concentrations varying substantially between individual sources within the same geographic area [43]. Nevertheless, reported well-water use provides no information about contaminant identity, concentration, treatment practices, duration of use, or actual intake. The observed difference could therefore reflect chemical or microbiological characteristics of individual sources, broader rural living conditions, or unmeasured behavioral and socioeconomic factors. It is more appropriately interpreted as supporting source-specific investigation than as evidence of an arsenic-related effect.

Occupational exposure to dust or smoke was also associated with symptom count in the mutually adjusted component model, whereas occupational chemical-agent contact and the remaining index components were not statistically supported. This result is difficult to interpret etiologically because the questionnaire did not document the specific agent, composition, concentration, frequency or duration of exposure, use of protective equipment, or temporal relationship with symptoms. Dust and smoke may represent highly variable mixtures, and the observed association may also reflect differences in occupation, working conditions, socioeconomic circumstances, or general health. The component-level results consequently reinforce the heterogeneous nature of the index rather than identifying a dominant exposure pathway.

At the symptom level, only metallic taste remained associated with the potential exposure index after correction for multiple comparisons. Metallic taste is nonspecific and may occur in a range of dental, medication-related, nutritional, clinical, and environmental contexts. The absence of statistically supported associations for the other individual symptoms after Holm correction, together with the lack of full covariate adjustment in the item-level models, argues against defining a characteristic symptom pattern. These results are therefore more useful for generating hypotheses than for identifying a contaminant-related clinical profile.

Biological mechanisms described in populations with confirmed chronic inorganic arsenic exposure provide relevant context but do not resolve this uncertainty. Arsenic toxicity has been associated with oxidative stress, disruption of thiol-dependent pathways, mitochondrial and endothelial dysfunction, inflammatory signaling, neurotoxicity, and epigenetic alterations [23,24]. These mechanisms may contribute to neurological and systemic manifestations when exposure has been adequately characterized, but mechanistic plausibility cannot establish that arsenic caused the nonspecific symptoms reported in the present survey. Contemporaneous household-water measurements, arsenic speciation, and biomarkers of internal exposure would be required to examine such an interpretation more directly.

From a public-health perspective, the potential value of combining historical environmental information with participant-reported characteristics lies primarily in prioritizing further investigation rather than classifying individual risk. Given the modest magnitude of the observed association, these findings should be regarded as hypothesis-generating and as providing preliminary contextual information rather than a basis for population-level screening decisions. Area-level monitoring can identify communities in which contamination has previously been documented, while questionnaire data may indicate whether residents continue to rely on potentially vulnerable water sources or report other circumstances relevant to exposure assessment. Considered alongside independent environmental evidence, these sources may help inform decisions about whether updated environmental surveillance or confirmatory household-water testing would be useful. They cannot establish which individuals have been exposed or require clinical assessment, but they may help identify where more specific investigation would be informative.

This distinction is especially relevant for decentralized and private water supplies, for which safety cannot be inferred from geographic location or source type alone. WHO emphasizes the screening and testing of drinking-water supplies and the provision of safe alternative sources where arsenic concentrations exceed applicable limits [43]. Guidance for private wells similarly emphasizes direct testing because contaminant presence and concentration cannot be determined without analyzing the individual source [44]. Where exceedances are confirmed, public-health action should therefore proceed from source-specific measurement to exposure reduction and access to an appropriate alternative water supply. Biomonitoring may subsequently be useful in appropriately designed investigations to characterize internal exposure, but it should complement rather than replace environmental source assessment [40].

Several features strengthen the interpretability of the study. The analysis brought together two clearly differentiated sources of information: previously published sample-level environmental measurements and participant-reported characteristics collected in the current survey. The historical arsenic classification was linked transparently through standardized locality clusters, while the questionnaire-derived index was analyzed continuously and examined across several alternative specifications. The primary analysis retained the full symptom count, accounted for overdispersion and within-locality clustering, and incorporated relevant demographic, behavioral, and health-related covariates. The concordant direction of the count-based and logistic sensitivity estimates, together with the component-level and item-level analyses, provides a broader view of the observed associations than reliance on a single model or an arbitrary binary threshold.

These strengths should be considered alongside important limitations. The cross-sectional design does not establish whether the reported potential-exposure characteristics preceded the symptoms, and reverse causality remains possible. Symptoms or pre-existing health concerns may have influenced water-source choices, reported behaviors, healthcare contact, or willingness to participate. Recruitment was convenience-based, the sample differed demographically from the county population, and volunteer and non-response bias could not be quantified. Individuals concerned about environmental contamination or their health may have been more likely to participate, whereas people with lower perceived relevance, more limited institutional contact, or other barriers may have been underrepresented.

Exposure assessment was limited by the area-level linkage and the interval between environmental sampling and questionnaire administration. No contemporaneous household-water measurements, arsenic speciation, or biomarkers were available to characterize current individual exposure. The potential exposure index was a nonvalidated formative summary of heterogeneous self-reported characteristics, and its simple additive construction did not account for differences in exposure intensity, duration, chemical form, or toxicological relevance. The symptom count likewise combined heterogeneous, nonspecific complaints without clinical confirmation or psychometric validation. Self-report and recall error may have affected both exposure-related variables and symptoms. Although the models adjusted for measured demographic, behavioral, and comorbidity variables, residual confounding by medication use, socioeconomic circumstances, psychosocial stress, occupational exposure intensity, detailed dietary patterns, and other environmental agents cannot be excluded.

Overall, the findings suggest that historical area-level monitoring and participant-reported characteristics may offer different but potentially complementary perspectives on environmental exposure. In the present study, the two sources were related to some extent, but they were not interchangeable and neither provided a direct measure of individual arsenic exposure. Their combined use may nevertheless provide preliminary contextual information that, together with independent environmental evidence, could help inform the design of subsequent confirmatory investigations. The contribution of this study lies primarily in illustrating how these two levels of information can be considered together during the early stages of environmental-health investigation, rather than in identifying a contaminant-specific symptom pattern or defining populations requiring surveillance.

## 5. Conclusions

In this community-based cross-sectional study, the questionnaire-derived potential exposure index was modestly associated with self-reported symptom count after adjustment for measured covariates and locality clustering, whereas the historical area-level arsenic screening classification was not. The direction of the association was generally consistent across alternative index specifications, although the component-level findings were heterogeneous and the drinking-water-focused estimate was smaller and less precise.

These results should be regarded as hypothesis-generating. Neither the historical screening classification nor the questionnaire-derived index provided a direct measure of individual arsenic exposure, and the observed associations cannot be attributed to arsenic or another specific contaminant. Future studies should combine contemporaneous household-water measurements, arsenic speciation, biomonitoring, clinically validated outcomes, and longitudinal or probability-based designs before individual or contaminant-specific inferences are made.

## Figures and Tables

**Table 1:** Domains and variables included in the study questionnaire.

Domain	Variables Assessed
Sociodemographic characteristics	Age, sex, locality of residence, duration of residence, and occupation
Environmental and occupational potential-exposure characteristics.	Primary drinking water source, use of water for drinking and cooking, daily water consumption, occupational exposure to dust, smoke, strong odors and chemical agents, presence of dental amalgam restorations
Lifestyle-related characteristics	Smoking status, alcohol consumption, dietary supplement and herbal product use, processed food consumption, fish consumption frequency
Self-reported symptom and health variables	Allergies or intolerances, skin problems, metallic taste, numbness or tingling sensations, memory or concentration difficulties, sleep disturbances, frequent headaches, irritability or mood changes, selected self-reported physician-diagnosed chronic conditions
Perception of drinking water quality	Perceived quality and safety of the primary drinking water source and willingness to change the water source if contamination was identified

**Table 2:** Environmental monitoring and arsenic HRI inputs underlying the historical area-level screening classification [25].

Parameter	Source-Study Specification	Use or Interpretation in the Present Study
Monitoring campaign	2022–2024; 271 drinking-water samples from 122 localities	Historical environmental context only
Recorded source type	181 centralized public-system samples; 15 private-source samples; 2 system entry/exit samples; 73 unspecified	No assumption that every participant used the historically sampled source
Arsenic measurements	Available for 244 samples; range 0.075–320.50 µg/L	No participant-specific concentration assigned
Adult CDI	CDI = C × IR/BW; IR = 2 L/day; BW = 70 kg	Calculated entirely at sample level in the preceding study
Adult arsenic HRI	HRI = CDI/RfD; source-study RfD = 0.0003 mg/kg/day	Historical screening calculation
Source-study adult HRI results	Mean 0.628; maximum 30.524; 14/244 samples > 1	Sample-level results, not individual participant risk estimates
Participant linkage	Adult HRI > 1 identified in 14 sample-level records from a limited number of localities; repeated records for the same locality retained separately in the source study	Participants residing in a locality represented by at least one adult HRI > 1 sample were assigned to the historical HRI ≥ 1 category; 7 standardized clusters and 91 participants were classified as HRI ≥ 1
Temporal correspondence	Environmental measurements, 2022–2024; questionnaire, November 2025–May 2026	Approximate interval of 1–4 years; source and concentration stability not verified
Current authoritative value	US EPA IRIS 2025 inorganic-arsenic RfD = 0.00006 mg/kg/day [37]	Historical classification retained for source-study consistency; not interpreted as a current classification under the updated RfD

CDI, chronic daily intake; HRI, Health Risk Index; IR, ingestion rate; BW, body weight; RfD, reference dose. Environmental measurements and HRI calculations were performed in the preceding study [25]. The present questionnaire study did not collect water samples or assign concentrations or doses to individual participants.

**Table 3:** Area-level screening classification, potential exposure index, and symptom outcomes.

Variable	Components	Coding/Range
Historical area-level arsenic screening classification	Historical binary area-level screening classification derived from the preceding environmental dataset and linked through the standardized locality cluster	HRI < 1 versus HRI ≥ 1; contextual area-level indicator, not participant-specific exposure
Potential exposure index	Seven binary indicators: well water, rural residence, water intake ≥ 2 L/day, occupational chemicals, dust/smoke, dental amalgam, and frequent fish; 1 point each	Theoretical 0–7; observed 0–6; analyzed continuously; no validated category thresholds
Symptom count	Eight heterogeneous, nonspecific self-reported symptoms	Theoretical 0–8; observed 0–7; primary count outcome
Binary symptom outcome	Derived from symptom count for sensitivity analysis	<3 vs. ≥3 symptoms; analytical, not clinical threshold

**Table 4:** Characteristics of the study population and exposure-related variables (N = 275).

Variable	Value
Age, years, mean ± SD	49.6 ± 14.9
Female sex, n (%)	169 (61.5)
Rural residence, n (%)	187 (68.0)
Historical area-level arsenic screening classification HRI ≥ 1, n (%)	91 (33.1)
Primary drinking water source, n (%)	
Tap water	72 (26.2)
Filtered water	39 (14.2)
Bottled water	83 (30.2)
Well water	81 (29.5)
Water consumption ≥ 2 L/day, n (%)	79 (28.7)
Dental amalgam restorations, n (%)	97 (35.3)
Smoking status, n (%)	
Current smokers	68 (24.7)
Former smokers	32 (11.6)
Never smokers	175 (63.6)
Potential exposure index	
Mean ± SD	2.20 ± 1.35
Median (IQR)	2 (1–3)
Observed range	0–6
Self-reported symptom count	
Mean ± SD	1.71 ± 1.67
Median (IQR)	1 (0–3)
Symptom count ≥ 3, n (%)	82 (29.8)

**Table 5:** Item-specific exploratory associations between the questionnaire-derived potential exposure index and self-reported symptoms.

Symptom	Events, n	OR per Point	95% CI	Raw *p*	Holm-Adjusted *p*
Allergies/intolerances	35	1.055	0.833–1.336	0.657	0.864
Skin problems	43	1.513	1.090–2.102	0.013	0.094
Metallic taste	21	1.798	1.223–2.642	0.003	0.023
Numbness/tingling	86	1.179	0.937–1.484	0.160	0.535
Memory/concentration difficulties	70	1.207	0.994–1.466	0.058	0.290
Sleep disturbances	104	1.128	1.001–1.271	0.048	0.289
Frequent headaches	47	1.175	0.952–1.451	0.134	0.535
Irritability/mood changes	63	1.087	0.883–1.336	0.432	0.864

OR, odds ratio; CI, confidence interval. Each row represents a separate logistic regression model with the potential exposure index entered continuously and finite-sample-corrected standard errors clustered by locality. The models were not adjusted for additional covariates. Holm-adjusted *p* values control the family-wise error rate across the eight symptom-specific tests. These analyses were exploratory. The symptoms were self-reported, nonspecific, and not clinically confirmed, and the results should not be interpreted as contaminant-specific or causal associations.

**Table 6:** Multivariable associations with self-reported symptom burden in the primary count-based model and the analytical binary sensitivity model (N = 275; 37 locality clusters).

Predictor	Primary Negative Binomial Model			Logistic Sensitivity Model: ≥3 Symptoms		
	Adjusted IRR	95% CI	*p*	Adjusted OR	95% CI	*p*
Intercept	0.314	0.184–0.535	<0.001	0.020	0.004–0.099	<0.001
Potential exposure index, per point	1.106	1.031–1.186	0.005	1.349	1.089–1.672	0.006
Age, per 10 years	1.134	1.055–1.217	<0.001	1.282	1.025–1.605	0.030
Female sex vs. male	1.821	1.410–2.351	<0.001	2.599	1.379–4.900	0.003
Current smoker vs. never	1.037	0.823–1.306	0.757	1.379	0.707–2.689	0.345
Former smoker vs. never	1.368	0.952–1.966	0.090	2.685	1.079–6.681	0.034
Any alcohol consumption vs. none	1.204	1.006–1.441	0.042	0.946	0.487–1.839	0.871
Historical HRI ≥ 1 vs. HRI < 1	1.135	0.829–1.554	0.431	0.946	0.461–1.941	0.881
Any self-reported comorbidity vs. none	1.798	1.498–2.160	<0.001	2.231	1.271–3.918	0.005

IRR, incidence rate ratio; OR, odds ratio; CI, confidence interval; HRI, Health Risk Index. The primary model was a negative binomial log-link regression of the complete self-reported symptom count. The logistic model used the analytical ≥3-symptom grouping as a secondary sensitivity outcome; this threshold has no validated clinical interpretation. Both models included the same prespecified predictors and used finite-sample-corrected standard errors clustered by locality. Reference categories were male sex, never smoking, no alcohol consumption, historical HRI < 1, and no self-reported comorbidity. All prespecified predictors are shown. IRRs and ORs quantify different outcome scales and should not be compared directly by magnitude.

**Table 7:** Alternative specifications of the questionnaire-derived potential exposure index and mutually adjusted component-level associations with symptom count.

Analysis/Specification	Adjusted IRR	95% CI	*p*
Alternative index specifications, per SD increase			
Primary equally weighted index	1.146	1.043–1.259	0.005
Alternative weighted specification	1.134	1.033–1.244	0.008
Drinking-water-focused specification	1.076	0.992–1.166	0.076
Index excluding rural residence	1.170	1.058–1.295	0.002
Index excluding dental amalgam and frequent fish consumption	1.109	1.004–1.225	0.042
Mutually adjusted individual components			
Well-water use	1.282	1.044–1.574	0.018
Rural residence	0.856	0.662–1.106	0.235
Daily water intake ≥ 2 L	0.936	0.738–1.188	0.588
Occupational chemical-agent contact	1.161	0.775–1.739	0.468
Occupational dust/smoke exposure	1.204	1.007–1.441	0.042
Dental amalgam currently present	1.251	0.984–1.591	0.068
Fish consumption ≥ 3 times/week	1.075	0.781–1.479	0.658

IRR, incidence rate ratio; CI, confidence interval; SD, standard deviation. Estimates for the alternative index specifications are reported per one population-standard-deviation increase to permit comparison across differently scaled indices. The primary index assigned one point to each of the seven binary indicators. The alternative weighted specification assigned three points to well-water use, two points to rural residence, and one point to each remaining indicator. The drinking-water-focused specification included well-water use and daily water intake ≥ 2 L. All models were adjusted for age, sex, smoking status, alcohol consumption, historical area-level arsenic screening classification, and self-reported comorbidity and used finite-sample-corrected standard errors clustered by locality. In the component-level model, all seven components were entered simultaneously. Equal numerical weighting does not imply equal exposure intensity, dose, chemical form, toxicological potency, or health risk. Component-level associations are exploratory and do not identify a contaminant or exposure pathway.

## Data Availability

The data presented in this study are available on request from the corresponding author. The datasets presented in this article are not readily available because the data are part of an ongoing doctoral research project and contain participant-level health and geographic information. Deidentified participant-level data, variable definitions, and statistical analysis code may be made available upon reasonable request to the corresponding authors.

## Supplementary Materials

The following supporting information can be downloaded at https://www.mdpi.com/article/10.3390/jox16040152/s1: Supplementary Material S1, Questionnaire Used for Environmental Exposure and Symptom Assessment; Table S1, Historical area-level arsenic screening classification, analyzed participant counts, administrative locality type, and reported well-water use across the 37 standardized locality clusters; Table S2, Published analytical context for potentially toxic elements in drinking water, Satu Mare County, 2022–2024 [16].

## Abbreviations

The following abbreviations are used in this manuscript:

AUC	Area Under the Receiver Operating Characteristic Curve
CI	Confidence Interval
HRI	Health Risk Index
IQR	Interquartile Range
OR	Odds Ratio
ROS	Reactive Oxygen Species
SD	Standard Deviation
US EPA	United States Environmental Protection Agency
VIF	Variance Inflation Factor
WHO	World Health Organization
AIC	Akaike Information Criterion
CDI	Chronic Daily Intake
IRR	Incidence Rate Ratio
NB	Negative Binomial
RfD	Reference Dose
