# Supplementary material for: Potential Exposure Characteristics and Self-Reported Symptom Burden in a Romanian Region with Heterogeneous Arsenic-Related Drinking-Water Risk
Source: J Xenobiot. 2026 Aug 15;16(4):152. doi: 10.3390/jox16040152 (PMC13514387; doi:10.3390/jox16040152)
Supplement: Supplementary file 1 [file jox-16-00152-s001.zip › Supplementary Material S1.pdf]

Supplementary Materials: Potential Exposure Characteristics and Self-Reported Symptom Burden in a Romanian Region with Heterogeneous Arsenic-Related Drinking-Water Risk

Dana Claudia Filipoiu, Daniela Gitea, Manuela Bianca Pasca, Gabriela S. Bungau, Ada Radu, Raul Ștefan-Pantiș , Alin Mogoș, Bogdan Uivaraseanu and Delia Mirela Tit

Questionnaire Used for Environmental Exposure and Symptom Assessment

INFORMATION FOR PARTICIPANTS

This questionnaire is part of a research project designed to investigate potential environmental exposure to trace metals through drinking water and lifestyle-related factors. The study aims to explore environmental exposure pathways and possible health effects associated with long-term exposure to environmental contaminants.

Participation is voluntary. Responses are anonymous and will be used exclusively for scientific research purposes. Completion of the questionnaire requires approximately 10–15 minutes.

Confidentiality

All information provided will be treated confidentially and analyzed anonymously. Participation is entirely voluntary, and refusal to participate will have no consequences of any kind.

Informed Consent

By completing this questionnaire, you confirm that:

- You have understood the purpose of the study.
- You agree to participate voluntarily.
- The information provided will be used solely for scientific purposes.
- You may withdraw from participation at any time without consequences.

SECTION A. SOCIODEMOGRAPHIC CHARACTERISTICS

1. Age

______ years

2. Sex

☐ Male

☐ Female

3. Place of residence

☐ Urban area

☐ Rural area

Locality: ______________________

4. How long have you lived in this area?

☐ <1 year

☐ 1–5 years

☐ 6–10 years

☐ >10 years

5. Occupation

☐ Farmer / agricultural worker

☐ Manual worker (factory, construction, warehouse)

☐ Driver / transportation worker

☐ Retail worker / salesperson

☐ Cleaning or caregiving worker

☐ Office employee
☐ Healthcare professional / pharmacist
☐ Teacher / educator
☐ Student
☐ Retired
☐ Currently unemployed
☐ Other: ______________________

SECTION B. WATER SOURCE AND ENVIRONMENTAL EXPOSURES

6. At your workplace, are you exposed to any of the following?
☐ Dust
☐ Smoke
☐ Strong odors
☐ Chemical substances
☐ None of the above / Do not know

7. Do you have dental amalgam fillings?
☐ No
☐ Yes, currently present
☐ Yes, but they have been removed

8. What is your primary source of drinking water?
☐ Tap water
☐ Filtered water
☐ Bottled water
☐ Well water / private well

9. Do you use this water source for:
Drinking: ☐ Yes ☐ No
Cooking: ☐ Yes ☐ No

10. Approximate daily water consumption
☐ <1 L/day
☐ 1–2 L/day
☐ 2–3 L/day
☐ >3 L/day

SECTION C. LIFESTYLE FACTORS

11. Smoking status
☐ Never smoker
☐ Current smoker
☐ Former smoker

12. Alcohol consumption
☐ Never
☐ Occasionally
☐ Frequently
☐ Daily

13. Do you use dietary supplements, herbal products, or natural remedies?
☐ No
☐ Yes

14. Consumption of fast food or processed foods
☐ Never
☐ Occasionally
☐ Weekly
☐ Daily

15. Fish consumption frequency
☐ Never
☐ 1–2 times per week
☐ ≥3 times per week

SECTION D. HEALTH STATUS

16. Do you have allergies or food intolerances?
☐ Yes
☐ No

17. Do you experience skin problems (itching, rashes, irritation)?
☐ Yes
☐ No

18. Do you experience a metallic taste in your mouth?
☐ Yes
☐ No

19. Do you experience numbness or tingling sensations in your hands or feet?
☐ Yes
☐ No

20. Do you experience difficulties with memory or concentration?
☐ Yes
☐ No

21. Do you experience sleep disturbances (insomnia or frequent awakenings)?
☐ Yes
☐ No

22. Do you experience frequent headaches?
☐ Yes
☐ No

23. Do you experience mood changes or irritability?
☐ Yes
☐ No

24. Have you ever been diagnosed by a healthcare professional with any of the following conditions?
☐ Cardiovascular disease (e.g., hypertension, arrhythmias)
☐ Diabetes mellitus
☐ Kidney disease
☐ Neurological disease (e.g., Parkinson’s disease, neuropathies)
☐ Thyroid disorders
☐ Other: ______________________
☐ No known medical conditions

SECTION E. PERCEPTION OF DRINKING WATER QUALITY

25. How would you rate the quality of the water you usually consume?
☐ Good
☐ Unsafe
☐ Do not know

26. If you were informed that your drinking water source was contaminated, would you change it?
☐ Yes
☐ No
☐ Not sure

Note. The original questionnaire was administered in Romanian. The present English version is provided for transparency, reproducibility, and methodological documentation purposes.
