# Supplementary material for: Potential Exposure Characteristics and Self-Reported Symptom Burden in a Romanian Region with Heterogeneous Arsenic-Related Drinking-Water Risk
Source: J Xenobiot. 2026 Aug 15;16(4):152. doi: 10.3390/jox16040152 (PMC13514387; doi:10.3390/jox16040152)
Supplement: Supplementary file 1 [file jox-16-00152-s001.zip › Supplementary Material S2.pdf]

Supplementary Materials: Potential Exposure Characteristics and Self-Reported Symptom Burden in a Romanian Region with Heterogeneous Arsenic-Related Drinking-Water Risk

Dana Claudia Filipoiu, Daniela Gitea, Manuela Bianca Pasca, Gabriela S. Bungau, Ada Radu, Raul Ștefan-Pantiș , Alin Mogoș, Bogdan Uivaraseanu and Delia Mirela Tit

Table S1. Historical area-level arsenic screening classification, analyzed participant counts, administrative locality type, and reported well-water use across the 37 standardized locality clusters.

Cluster ID	Standardized locality	Historical area-level arsenic screening classification	Analyzed participants, n	Rural locality	Well-water users, n
L01	Agris	HRI < 1	6	Yes	2
L03	Babasesti	HRI < 1	1	Yes	0
L04	Berveni	HRI < 1	5	Yes	1
L05	Bixad	HRI < 1	1	Yes	1
L06	Calinesti Oas	HRI < 1	5	Yes	3
L07	Carei	HRI < 1	18	No	3
L08	Chereusa	HRI < 1	1	Yes	1
L09	Dacia	HRI < 1	1	Yes	0
L10	Decebal	HRI < 1	3	Yes	0
L11	Dindesti	HRI < 1	8	Yes	5
L12	Doba	HRI < 1	9	Yes	0
L13	Draguseni	HRI < 1	7	Yes	3
L14	Giorocuta	HRI < 1	1	Yes	0
L15	Hurezu Mare	HRI < 1	1	Yes	0
L16	Iegheriste	HRI < 1	3	Yes	0
L17	Lucaceni	HRI < 1	1	Yes	0
L18	Madaras	HRI < 1	1	Yes	0
L19	Mediesu Aurit	HRI < 1	6	Yes	2
L20	Micula	HRI < 1	4	Yes	2
L21	Negresti Oas	HRI < 1	8	No	1
L22	Paulian	HRI < 1	1	Yes	0
L26	Poiana Codrului	HRI < 1	6	Yes	3
L27	Potau	HRI < 1	2	Yes	2
L28	Racova	HRI < 1	1	Yes	0
L29	Remetea Oas	HRI < 1	2	Yes	2
L31	Rona de Jos	HRI < 1	1	Yes	0

L32	Satu Mare	HRI < 1	56	No	2
L34	Supuru de Jos	HRI < 1	6	Yes	0
L36	Turt	HRI < 1	7	Yes	5
L37	Viile Satu Mare	HRI < 1	12	Yes	6
L02	Andrid	HRI ≥ 1	8	Yes	5
L23	Petresti	HRI ≥ 1	28	Yes	10
L24	Pir	HRI ≥ 1	2	Yes	2
L25	Piscolt	HRI ≥ 1	43	Yes	17
L30	Resighea	HRI ≥ 1	2	Yes	0
L33	Scarisoara Noua	HRI ≥ 1	1	Yes	0
L35	Tasnad	HRI ≥ 1	7	No	3

HRI, Health Risk Index. The historical HRI classification was derived from the preceding environmental study and linked through standardized locality clusters. It represents area-level historical screening context and does not indicate participant-specific water concentration, exposure, or dose. Administrative locality type describes the standardized locality cluster and does not necessarily reproduce the participant-level rural-residence coding used in the analytical dataset. In the individual-level dataset, 187 participants were coded as rural and 88 as urban; these values were used in construction of the potential exposure index and in all statistical analyses. Locality-specific questionnaire distribution and response counts were unavailable; therefore, response rates by locality could not be calculated.

Table S2. Published analytical context for potentially toxic elements in drinking water, Satu Mare County, 2022–2024 [16]

Element	n	CMA (μg/L)	Observed range (μg/L)	Source-study interpretation
As	244	10	0.075–320.50	Localized exceedances; principal source-study contaminant of concern.
Cd	217	5	0.04–3.05	All observed values below CMA.
Cr (total)	217	50	0.075–29.70	All observed values below CMA; speciation unavailable.
Mn	142	50	0.38–460.70	Repeated rural exceedances during 2023–2024.
Ni	217	20	0.38–31.50	Predominantly low values with an isolated maximum above CMA.
Pb	217	10	0.38–7.60	All observed values below CMA.
Se	157	20	0.76–33.00	Predominantly low/censored values with isolated maxima above CMA.

CMA, Romanian maximum admissible concentration reported in the source study under Ordinance 7/2023. Analytical coverage varied by element. The table summarizes measurements published previously [16]; no water samples were collected in the questionnaire study, and only the historical arsenic HRI classification was linked to participants.
